# Supplementary material for: Interface-dominated sliding compound drops
Source: Eur Phys J E Soft Matter. 2026 Aug 10;49(8):72. doi: 10.1140/epje/s10189-026-00612-9 (PMC13457257; doi:10.1140/epje/s10189-026-00612-9)
Supplement: Supplementary file 1 [file 10189_2026_612_MOESM6_ESM.pdf]

Supplementary Material: Overview

TABLE I: This table is meant to give an overview of the Supplementary Material that is provided with "Interface-dominated sliding compound drops". Note that here only the three parameters (β, ν, η) varied across the study are shown, other parameters are given in section 2.2. The videos are computed in the frame moving with the center-of-mass (COM) velocity U of the compound drop. Note that the time resets after initial relaxation when the inclination is applied in each run except for movie 2, see description below.

**File name**	**Description**	**Relevant parameters**
movie1_ThDT2026_beta_cfg_ch	Configuration change (from 2-1 to 1-2) induced by exceeding $\beta_{\mathrm{c}21}$, [Fig. 3(a)] .	(β, ν, η) = $(2.16 \times 10^{-5}, 1, 1)$
movie2_ThDT2026_per_splitting	Periodic dynamics of overtaking, fusing, and splitting compound drops occuring when exceeding $\beta_{\mathrm{c}12}$, [Fig. 3(a)]. Note that the frame is comoving with the COM velocity of liquid 1.	(β, ν, η) = $(2.65 \times 10^{-5}, 1, 5.5)$
movie3_ThDT2026_sliding-on-layer	Dewetting and coarsening of two initially flat liquids on an inclined substrate yielding a droplet sliding on a liquid substrate, [Fig. 3(a) and (h)]. Note that the frame is comoving with the COM velocity of liquid 2 and $\beta \neq 0$ from the beginning.	(β, ν, η) = $(1.3 \times 10^{-5}, 1, 1)$
movie4_ThDT2026_eta_cfg_ch	Configuration change (from 2-1 to 1-2) induced by choosing $\eta < \eta_{\mathrm{c}21}$, [Fig. 9(a)].	(β, ν, η) = $(2.5 \times 10^{-5}, 1, 3.67)$
movie5_ThDT2026_inv_cfg_ch	Inverse configuration change (from 1-2 to 2-1) induced by exceeding $\eta_{\mathrm{c}21\mathrm{b}}$ considerably, [Fig. 9(a)].	(β, ν, η) = $(2.5 \times 10^{-5}, 1, 11)$
